# Children’s mental time travel during mind wandering

**DOI:** 10.3389/fpsyg.2014.00927

**Published:** 2014-08-21

**Authors:** Qun Ye, Xiaolan Song, Yi Zhang, Qinqin Wang

**Affiliations:** Department of Psychology, College of Education, Zhejiang Normal UniversityJinhua, China

**Keywords:** mental time travel, mind wandering, task-unrelated thought, spontaneous thought, prospective cognition

## Abstract

The prospective bias is a salient feature of mind wandering in healthy adults, yet little is known about the temporal focus of children’s mind wandering. In the present study, (I) we developed the temporal focus of mind wandering questionnaire for school-age children (TFMWQ-C), a 12-item scale with good test–retest reliability and construct validity. (II) The criterion validity was tested by thought sampling in both choice reaction time task and working memory task. A positive correlation was found between the temporal focus measured by the questionnaire and the one adopted during task-unrelated thoughts (TUTs) by thought sampling probes, especially in the trait level of future-oriented mind wandering. At the same time, children who experienced more TUTs tended to show worse behavioral performance during tasks. (III) The children in both tasks experienced more future-oriented TUTs than past-oriented ones, which was congruent with the results observed in adults; however, in contrast with previous research on adults, the prospective bias was not influenced by task demands. Together these results indicate that the prospective bias of mind wandering has emerged since the school-age (9∼13 years old), and that the relationship between mental time travel (MTT) during mind wandering and the use of cognitive resources differs between children and adults. Our study provides new insights into how this interesting feature of mind wandering may adaptively contribute to the development of children’s MTT.

## INTRODUCTION

Mental time travel (MTT) refers to “the faculty that allows humans to mentally project themselves backward in time to re-live, or forward to pre-live events” and plays a very important role in autonoetic consciousness that helps people to maintain a continuous feeling of “self” extending from the personal past through the present to the personal future (Tulving, 1985). Recent research has demonstrated that MTT can arise spontaneously or involuntarily (Berntsen and Jacobsen, 2008), that is, being initiated without any conscious act of the will. This phenomenon is labeled as spontaneous (involuntary) MTT, and has been found very common in everyday life (Berntsen, 1996; Rubin and Berntsen, 2009). Although a lot of research on the spontaneous MTT has been conducted with adults, little is known about children’s spontaneous MTT. Conducting such research on children is especially important given that the presence of MTT has been established in early childhood (Atance and O’Neill, 2005; Busby and Suddendorf, 2005).

Mind wandering refers to engagement in cognitions unrelated to the current demands of the external environments (Schooler et al., 2011), and constitutes as much as 50% of our waking thoughts (Killingsworth and Gilbert, 2010). Many researchers have suggested that the content of mind wandering shows remarkable temporal focus (Smallwood et al., 2009b; Song and Wang, 2012), suggesting that spontaneous MTT may be the major component in mind wandering. Moreover, it has been suggested that the variation in mind wandering represents an important individual difference (Kane et al., 2007; Mrazek et al., 2013; Unsworth and McMillan, 2014), and that this difference can also be reflected in its temporal focus. Yet so far to date, there was not a valid tool to measure the individual differences in spontaneous MTT.

Furthermore, the prospective bias (i.e., people are more inclined to experience future-oriented mind wandering rather than past-oriented mind wandering) of mind wandering is a salient feature in healthy adults, and has been observed in different populations (Smallwood et al., 2009b, 2011; Smallwood and O’Connor, 2011; Stawarczyk et al., 2011; Song and Wang, 2012). There is also some evidence to suggest that executive resources could support future-oriented mind wandering. Specifically, subjects with higher working memory capacity (WMC) tend to experience more future-oriented mind wandering than the ones with lower WMC (Baird et al., 2011), and the prospective bias of mind wandering was curtailed by the requirement to continuously monitor the task (Smallwood et al., 2009b). Such results could be explained by the argument that simulating the future recruits more cognitive resources than recalling the past (Addis et al., 2007; Szpunar et al., 2007). In the current research, the question is whether there are any prospective biases of mind wandering in children? If so, what is the relationship between different temporal focuses of children’s mind wandering and executive resources?

Given the above considerations, in study 1, we aimed to develop and validate a measuring tool – the temporal focus of mind wandering questionnaire for children (TFMWQ-C) – to explore the characteristics of children’s spontaneous MTT during mind wandering. And in study 2, we tested the criterion validity of the TFMWQ-C in two laboratory tasks with different working memory load, and explored how executive resources would influence the temporal focus of spontaneous MTT during mind wandering.

It has been suggested that the key developments of MTT occur between 3 and 5 years of age (Atance and O’Neill, 2005; Busby and Suddendorf, 2005; Atance, 2008b), and its neurophysiological foundations also need to develop throughout childhood (Atance, 2008a). According to our preliminary research, in which we interviewed 100 children about their understanding of their inner experiences, children could not describe their mind wandering experiences accurately by introspection until 8 years old (Chen, 2013). Consequently, we only included children ages 8 or older to participate in the present research.

## EXPERIMENT OVERVIEW

Two studies were conducted to investigate the characteristics of children’s spontaneous MTT during mind wandering. Study 1 developed the TFMWQ-C and determined its reliability and validity in a large sample of school-age children. Study 2 used a separate sample to explore the criterion validity of the TFMWQ-C by experience sampling method (ESM) in two tasks with different cognitive load, and so the association between the executive resources and the temporal focus of their spontaneous MTT for children could be explored.

## STUDY 1

In the current study, we aimed to develop and validate a questionnaire to measure the individual differences in the temporal focus during spontaneous MTT in daily life for primary school students. At the same time, the gender and grade differences in the temporal focus of spontaneous MTT were investigated.

### METHODS AND RESULTS

#### Participants

The first sample [for item development and exploratory factor analysis (EFA)] included 490 school-aged children from a primary school in East China (52.3% female, mean age = 10.94, range 8–14 years; Sample B+C, **Table 1**). The second sample [for confirmatory factor analysis (CFA)] included 250 school-aged children from the same school (54.4% female, mean age = 10.53, range 8–13 years; Sample D, **Table 1**). An independent sample of 66 students was recruited for test–retest reliability (54.5% female, mean age = 10.64, range 9–14 years; Sample E, **Table 1**).

**Table 1:** Sample characteristics.

Characteristic	Sample A	B	C	D	E	F
*N*	22	222	268	250	66	71
Age range (years)	6–13	9–14	8–13	8–13	9–14	9–13
Age mean (years)	9.14	11.26	10.67	10.53	10.64	11.4
Boy (%)	54.5	42.8	50.0	45.2	45.5	47.9
Girl (%)	45.5	56.3	48.9	54.4	54.5	52.1

The research procedure was in accordance with the ethical principle of the 1964 Declaration of Helsinki (World Medical Organization). The institute review board of Zhejiang Normal University approved the research procedure.

#### Interview

To facilitate the development of the item pool to the target population, we recruited 22 school-aged children from two primary schools for the pilot interview (see Sample A, **Table 1**). The main contents of the interview involved the interviewee’s understanding of mind wandering, and the content, temporal focus, frequency and emotional valence of their mind wandering experiences. We encouraged the interviewees to give several examples of their own mind wandering experiences. Each interview was conducted one on one and lasted for about 20 min. Because the main goal of the current study was to assess children’s MTT during mind wandering, only the results about the temporal focus were described here.

The participants’ responses in the interview showed that the children age 8 and older were very familiar with their mind wandering experiences, and had no difficulty in giving examples and reflecting on mind wandering episodes in their daily lives. As soon as children showed the capacity to generate these examples, tendencies to recall the past and to envision the future were both very common in their reports of mind wandering episodes.

#### Item development

The item pool was constructed based on the interview and the existing questionnaires about the daydreaming experiences for adults and children (Singer and Antrobus, 1972; Rosenfeld, 1979; Vooijs et al., 1992). After piloted item development with 222 participants (see Sample B, **Table 1** and expert reviewed system, 18 items were retained. A five-point response scale (1-strongly agree, 2-agree, 3-uncertain, 4-disagree, 5-strongly disagree) was used to promote adequate variance and scale reliability.

#### Exploratory factor analysis

The sample included 333 Chinese school-aged children, and 268 complete surveys were obtained with an efficient rate of 80.48% (see Sample C; **Table 1**). A principal component analysis (PCA) with oblique rotation reduced the 18 items to two factors, which accounted for 55.19% of the variance in the data. Six items were removed as they did not load strongly on one factor or had limited conceptual relevance to any particular factor. The two factors were labeled as “Future Orientation” (item 1, 3, 5, 7, 9, 11) and “Past Orientation” (item 2, 4, 6, 8, 10, 12) respectively. Inventory items and factor loadings are presented in **Table 2**. The Kaiser measure of sampling adequacy was 0.91, sharing common factors among these items. Bartlett’s test of Sphericity also indicated that the variable data was suitable for factor analysis.

**Table 2:** Pattern matrix factor loadings of the TFMWQ-C (oblique rotation method).

FMWQ-C items (item #; have been translated into English)	Factor loadings	
	F1 (future)	F2 (past)
I often imagine spontaneously what I will be doing a few years from now. (7)	**0.90**	-0.12
Ideas about the future often come into my mind suddenly. (3)	**0.80**	0.03
I often cannot help imagining what the world will be like in the future. (9)	**0.80**	-0.01
When daydreaming, I often imagine what I will be like when I grew up. (1)	**0.78**	-0.01
When mind wandering, I often think where I will go in a few years. (5)	**0.66**	0.04
I sometimes involuntarily think about where my good friends will go and what they will do in a few years. (11)	**0.54**	0.23
I am often suddenly reminded of things my parents or teachers said to me in my childhood. (2)	-0.05	**0.81**
I often involuntarily think about things that happened in my childhood. (4)	0.05	**0.73**
I sometimes recall memorable things that happened in the past. (10)	-0.01	**0.72**
Childhood playmates often suddenly appear in my mind. (8)	0.03	**0.70**
I often involuntarily recall children’s songs or stories my parents told me when I was a child. (12)	-0.03	**0.67**
I often involuntarily recall times when I was playing. (6)	0.06	**0.64**

Factor loadings exceeding 0.5 are highlighted.

#### Confirmatory factor analysis

We performed a CFA of the two-factor model in a new sample of 250 school-age children (see Sample D; **Table 1**), using maximum-likelihood estimation and the AMOS 4.0 (Arbuckle and Wothke, 1999). The correspondence between the fitted covariance matrix of the two-factor model and the sample covariance matrix of the actual data was tested by a number of fit indices. χ^2^ (a non-significant value corresponds to an acceptable fit), is known to be susceptible to estimate parameters and sample size and it has been emphasized that it is unusual to obtain a non-significant χ^2^ when performing CFA on self-report questionnaires (Byrne, 1994). In order to reduce the effect of sample size, it is generally considered that the ratio between chi-square and degrees of freedom <2 can be used to indicate the fitness of the model (Carmines and McIver, 1981). In addition, four indices of model fit were computed: the root mean square error of approximation (RMSEA), the standard root mean square residual (SRMR), the comparative fit index (CFI), and the goodness-of-fit index (GFI). These values all reached the ideal of the priori standard [χ^2^ (53) = 85.755, *p*< 0.05, χ^2^/df = 1.618, RMSEA = 0.050, SRMR = 0.046, CFI = 0.965, GFI = 0.944]. And a moderate degree of correlation between the two factors was revealed (*r*= 0.58, *p*< 0.001).

At the same time, all 12 items of the scale were significantly related to the latent factor (all *p*< .001) and the average value of these item’s factor loadings was 0.63. And the Cronbach’s Alpha value for TFMWQ-C was 0.86, indicating the satisfactory internal reliability of the scale. The combination of these indices indicated an acceptable fit.

#### Test–retest reliability

The temporal stability of the TFMWQ-C was examined in an independent sample (see Sample E, **Table 1**) over a 2 week period. Paired sample correlation analysis showed an excellent test–retest reliability of TFMWQ-C in school-age children (The Pearson *r* was 0.70 for Future Orientation, 0.78 for Past Orientation, and 0.80 for total score of TFMWQ-C respectively, *p*< 0.001).

#### Gender and grade differences

**FIGURE 1:**
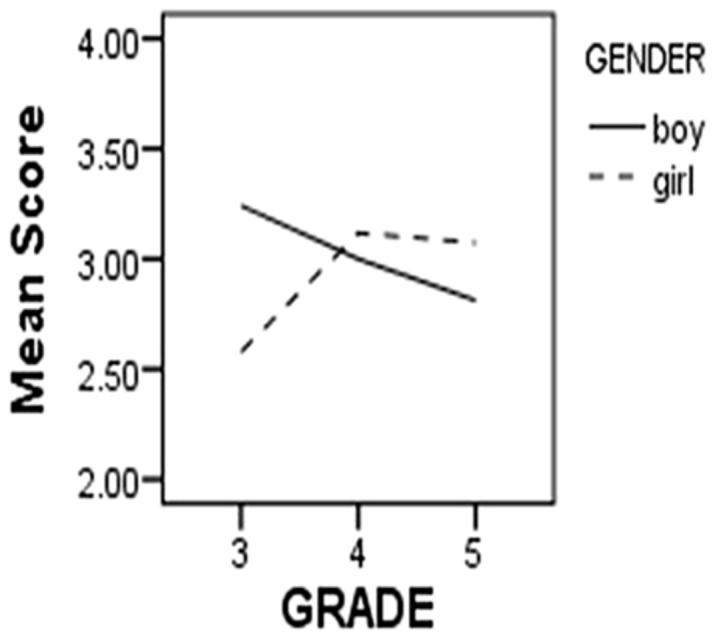
**Future dimension of children’s mind wandering**.

**FIGURE 2:**
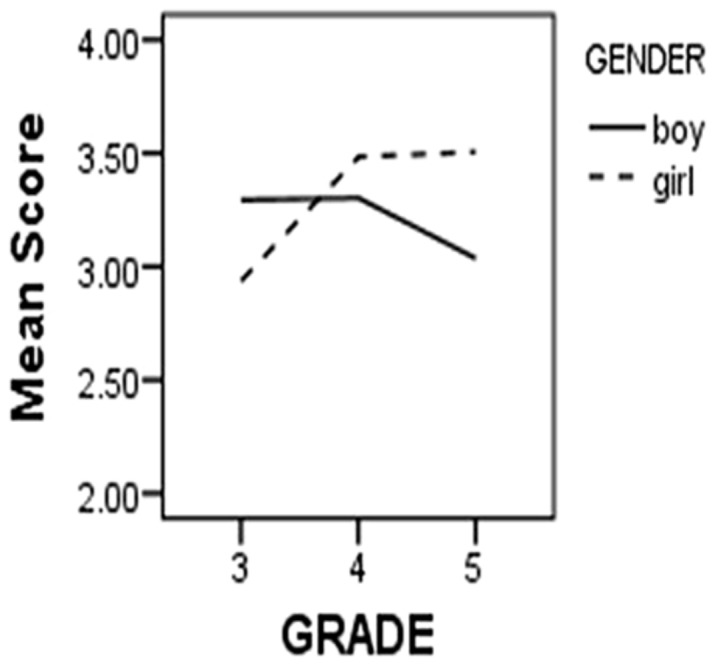
**Past dimension of children’s mind wandering**.

In this part, we looked at gender and grade differences in the TFMWQ-C based on the sample that the CFA was conducted on. A multivariate analysis of variances (ANOVA; dependent variable: average score in the Past Orientation and Future Orientation respectively) revealed no main effect of gender or grade, but a significant Gender × Grade interaction effect (Pillai’s Trace value was 0.05, *F*= 3.28, *p*< 0.05). To better understand the interaction effect, we performed univariate analysis on the variances of the two factors respectively (Past Orientation: interaction effect, *F*(2,244) = 5.17, *p*< 0.01; Future Orientation: interaction effect, *F*(2,244) = 4.14, *p*< 0.05). The simple main effects were further analyzed, and the results showed that for Future Orientation, the scores of girls varied across grades [*F*(2,244) = 3.45, *p*< 0.05], while those of boys did not [*F*(2,244) = 1.38, *p*= 0.26]. *Post hoc* LSD tests indicated that for girls, the scores on Future Orientation of the fourth and fifth grade were higher than that of the third grade (both *p*< 0.05), and no difference was observed between the fourth and fifth grade (*p*= 0.83). The results for the scores on Past Orientation were substantially the same with Future Orientation [Girl: *F*(2,244) = 6.63, *p*< 0.01; Boy:* F*(2,244) = 1.42, *p*= 0.24]. This interaction effect suggested that there was a significant increase between 3rd and 4th grade in past-oriented and future-oriented mind wandering, but only for girls (**Table 3**, **Figures 1 and 2**).

**Table 3:** Descriptive statistics of different temporal focus between gender and grade in the TFMWQ-C (*N* **=** 250).

Temporal focus	Gender	Grade	*M*	*SD*	*N*
Future	Boy	3	3.19	0.89	30
		4	3.00	0.90	38
		5	2.81	1.07	46
	Girl	3	2.58	0.93	34
		4	3.12	0.89	41
		5	3.07	1.11	61
Past	Boy	3	3.26	0.67	30
		4	3.30	0.72	38
		5	3.04	0.86	46
	Girl	3	2.94	0.78	34
		4	3.48	0.74	41
		5	3.51	0.81	61

### DISCUSSION

In study 1, we developed a questionnaire to assess MTT during mind wandering for school-age children. The resulting TFMWQ-C was shown to have a reliable two-factor structure, derived by EFA and confirmed by CFA, and was found to have satisfactory internal reliability and test–retest reliability. Our findings thus confirmed the suggestion that there are empirically determinable individual differences in MTT during mind wandering for school-age children.

It also showed that at least for school-aged children, the tendency to experience past-oriented MTT during mind wandering was positively correlated with the future-oriented counterpart, which suggests that the ability to simulate future events relies on many of the same cognitive and neural processes as remembering past events (Schacter and Addis, 2007; Schacter et al., 2007; Buckner, 2010; Richmond and Pan, 2013).

Another finding was the gender difference in the development of temporal oriented mind wandering, which suggests that there is a significant increase in the experiences of spontaneous MTT between 3rd and 4th grade for girls but not boys. This finding parallels other gender differences in cognitive abilities, such as differences in verbal ability, that emerge during the primary school years (Hyde and Linn, 1988).

## STUDY 2

Having established the construct validity and internal consistency reliability of TFMWQ-C in study 1, we next evaluated the criterion validity of TFMWQ-C in two laboratory tasks that were commonly used in mind wandering research [choice reaction time task (CRT) for study 2A and 1-back working memory task (WMT) for study 2B] by examining the correlation between the participants’ scores on TFMWQ-C and their frequencies of past and future-oriented MTT during mind wandering. In these two tasks a thought sampling method was used to catch participants’ spontaneous episodes of mind wandering. This method was the most widely used one in assessing mind wandering, involving periodically interrupting participants by thought probes during a task at unpredicted occasions and asking them to report whether their real-time inner experience was on the task or task-unrelated thoughts (TUTs; Giambra, 1995; Teasdale et al., 1995; Schooler et al., 2004). There was a broad literature validating the self-reported mind wandering obtained through thought sampling method in a variety of task situations among adolescents and adults (Smallwood and Schooler, 2006; Smallwood et al., 2008; Christoff et al., 2009; Killingsworth and Gilbert, 2010; Mrazek et al., 2013).

Another goal of Study 2 was to explore the temporal focus of children’s spontaneous MTT during laboratory tasks. Although the prospective bias of mind wandering in different situations has been observed across different populations among adults (Smallwood et al., 2009b, 2011; Stawarczyk et al., 2011; Song and Wang, 2012), little is known about whether it is the same for children.

For adult participants, previous research demonstrated that future-oriented thinking was more prevalent in CRT tasks than in WMT (Smallwood et al., 2009b). In addition, some researchers have argued that working memory load should disproportionately reduce the amount of prospective thought relative to retrospective thought in adults (Smallwood et al., 2009b, 2011). Therefore the third goal of this study was to explore whether the availability of executive resources influences the prospective and retrospective focus during mind wandering for children in the same way as adults. CRT and WMT are two tasks commonly used for investigating the relation between executive resources and prospective bias of mind wandering (Smallwood et al., 2009b, 2011), and they differ on the need to recruit executive resources. CRT merely requires the subjects to wait for a color number to occur and judge whether the number is odd or even, while WMT makes the subjects keep the recent numbers in mind and judge whether previous number was odd or even. Here study 2A adopted CRT and study 2B adopted WMT.

## STUDY 2A

### MATERIALS AND METHODS

#### Participants

Seventy-one school-age children completed this experiment (see Sample F; **Table 1**). All participants had normal or corrected to normal vision. Two participants were excluded from the analysis as their accuracy rates in CRT exceeded 3 SD from average.

#### Procedure

Participants first completed an adapted version of CRT (Smallwood et al., 2009b) and then completed the TFMWQ-C at a computer. At the end, the participants received a gift for their participation.

#### Choice reaction time task

Stimuli for CRT were numeric digits, 1–9, which were constituted by 190 frequent non-target numbers (colored black) and 24 infrequent target numbers (colored green) in a white background with a quasi-random order of presentation. Stimulus presentation rate was 1 item every 2000 ms (followed by 1000 ms fixation cross). Participants were required to make a decision about whether an infrequent number was odd or even using the computer keyboard (F for the odd, J for the even). The stimuli were presented using E-Prime presentation software on a computer (Schneider et al., 2002). The testing session for this task lasted approximately 15 min.

#### Thought probes

During the experiment, participants wore headphones. At six different pseudo-random occasions a “ding” sound suddenly appeared via the headphone with a prompt screen showing the thought sampling question with three options: “Just in the moments prior to the probe, what were you thinking about? 1-Thinking about something from the past,or 2-Just being on the task or 3-Thinking about something from the future.” Participants made responses by keyboard. Before the task, the participants received instructions and examples explaining the different options to ensure all of them understood the question.

### RESULTS AND DISCUSSION

Participants maintained reasonable accuracy throughout the task (*M*= 0.83, SE = 0.01). Next we considered the correspondence between the trait level and the state level of the temporal focus in spontaneous MTT by computing the correlation between the scores on TFMWQ-C and the frequencies of future/past-oriented mind wandering during laboratory tasks. The results showed a moderate positive correlation between the average total score in TFMWQ-C and the frequencies of TUT in CRT (*r*= 0.26, *p*< 0.01), indicating that the participants who reported more spontaneous MTT in their daily lives also reported more TUTs during CRT (**Table 4**). At the same time, the score on Future Orientation in TFMWQ-C was positively correlated with the frequency of future-oriented TUTs during CRT task, whereas there was no such correlation in Past Orientation. Another interesting finding was that participants experienced more future-oriented TUTs than past-oriented TUTs [*M_future_*= 0.25, *M_past_*= 0.14, *t*(68) = 3.93, *p*< 0.001]. These results provide evidence that the prospective bias of mind wandering during laboratory tasks that has been observed in adults is also present in school-age children.

**Table 4:** Means (*M*), standard deviations (*SD*), and correlations between TFMWQ-C and CRT.

	*M (SD)*	1	2	3	4	5
Q.F	3.48 (0.09)					
Q.P	3.63 (0.08)	0.19				
Q.FP	3.55 (0.07)	0.80**	0.74**			
CRT.F	0.25 (0.02)	0.34**	0.16	0.33**		
CRT.P	0.14 (0.02)	0.01	-0.02	-0.01	0.01	
CRT.FP	0.39 (0.03)	0.28*	0.11	0.26*	0.80**	0.61**

*N* = 69. Q.F, average score of Future Orientation in the questionnaire; Q.P, average score of Past Orientation in the questionnaire; Q.FP, average total score in the questionnaire; CRT.F, frequencies of future-oriented TUT in the CRT; CRT.P, frequencies of past-oriented TUT in the CRT; CRT.FP, frequencies of TUT in the CRT. *p < 0.05, **p < 0.01.

## STUDY 2B

### MATERIALS AND METHODS

The children who participated in study 2A completed the 1-back WMT 1 week later (see Sample F, **Table 1**). The procedure of the WMT was the same as study 2A except that here the participants were required to decide if the stimulus preceding the infrequent target (a green “?”) was odd or even (targets *N* = 24, non-targets *N* = 202, probe *N* = 6). The experiment lasted ∼13 min. After excluding two participants with very low accuracy rates and one participant with a software error from the analysis, there remained a total 66 valid data in both tasks.

### RESULTS AND DISCUSSION

Participants maintained similar reasonable accuracy rates throughout the task (*M*= 0.83, SE = 0.01), whereas it was not significantly different from that of CRT [*t*(65) = 0.11, *p*= 0.91]. However, reaction time in the WMT (*M* = 1279 ms, SE = 31.56) was significantly slower than the one in the CRT (*M* = 1002 ms, SE = 17.13),* t*(65) = 8.25,* p*< 0.001. From this perspective, the WMT indeed was more demanding than the CRT. As expected, TFMWQ-C scores also correlated with probe-caught TUTs during the WMT (*r*= 0.32, *p*< 0.01), and a positive correlation was found again between the temporal feature revealed by the questionnaire and TUTs during WMT, but this was also limited in the Future Orientation (**Table 5**). At the same time, the frequency of TUT during WMT was significantly associated with lower accuracy rate (*r*= -0.38, *p*< 0.01), which was consistent with the claim that mind wandering was always associated with poor performance during a highly demanding task.

**Table 5:** Means (*M*), standard deviations (*SD*), and correlations between TFMWQ-C and WMT.

	*M (SD)*	1	2	3	4	5
Q.F	3.47 (0.09)					
Q.P	3.61 (0.08)	0.16				
Q.FP	3.54 (0.07)	0.80**	0.73**			
WMT.F	0.26 (0.03)	0.27*	0.21	0.32**		
WMT.P	0.11 (0.02)	0.17	0.02	0.13	0.12	
WMT.FP	0.37 (0.04)	0.30*	0.18	0.32**	0.85**	0.63**

*N* = 68. Q.F, average score of Future Orientation in the questionnaire; Q.P, average score of Past Orientation in the questionnaire; Q.FP, average score in the questionnaire; WMT.F, frequencies of task-unrelated thought of Future Orientation in the WMT; WMT.P, frequencies of task-unrelated thought of Past Orientation in the WMT; WMT.FP, frequencies of task-unrelated thought in the WMT. *p < 0.05, **p < 0.01.

**FIGURE 3:**
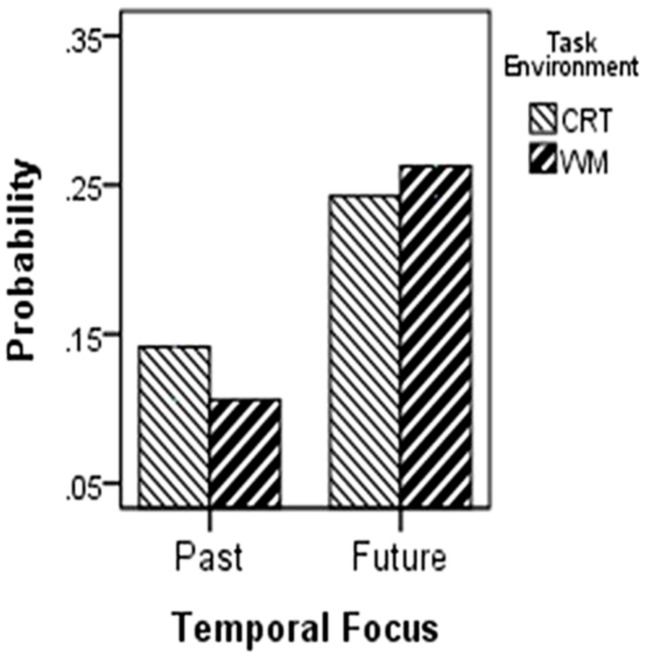
**2 (temporal focus: past, future) × 2 (task environment: CRT, WMT) repeated ANOVA**.

Combined with the results of study 2A, a highly positive correlation between the frequencies of TUT during the CRT and the WMT indicated a reasonable level of consistency in mind wandering across two different demanding contexts (*r*= 0.55, *p*< 0.001). Next, we considered how the task demands influenced the temporal focus of TUTs. A 2 (temporal focus: past, future) × 2 (task environment: CRT, WMT) repeated ANOVA yielded a main effect of temporal focus [*F*(1,65) = 29.93, *p*< 0.001, $\eta_{p}^{2}$= 0.315], indicating that future-oriented TUTs were more common than past-oriented TUTs. However, the main effect of task environment [*F*(1,65) = 0.22, *p*= 0.64, $\eta_{p}^{2}$ = 0.035] and the temporal focus × task environment interaction effect [*F*(1,65) = 1.89, *p*= 0.17, $\eta_{p}^{2}$ = 0.028] were not observed (**Figure 3**).

Children in both tasks tended to experience more future-oriented TUTs rather than past-oriented ones, as had been seen in adults. However, unlike adults, the prospective bias in children was not influenced by task demands (Smallwood et al., 2009b). There are two possible reasons for the result. One reason may lie in the fact that the participants did the CRT 1 week before the WMT, which made them more practiced in the WMT and reduced the difference of the executive load between the two tasks. Although the accuracy rate and the frequency of TUT indeed did not decrease significantly in the WMT, the longer RT suggested the higher executive load in WMT. Therefore the more likely explanation is that the relation between the temporal focus of MTT during mind wandering and the executive resources in children is different than in adults. Nevertheless, the prospective bias of mind wandering has emerged in school-age and the relation between different temporal focuses of mind wandering and executive resources deserves further investigation.

## GENERAL DISCUSSION

The studies presented in this article were designed to examine the temporal focus of children’s spontaneous MTT during mind wandering. In study 1, we used EFA and CFA to develop the TFMWQ-C, which contained two factors: past and future orientation. The TFMWQ-C was demonstrated to be a reliable and valid instrument for measuring the individual differences in spontaneous MTT for children from 3rd grade to 6th grade in primary school. Further validation of the TFMWQ-C is necessary across different cultures or special populations with extreme scores in the two temporal dimensions. For instance, specific deficits in prospective thought have been shown to increase suicide risk and undermine many adaptive cognitive functions (O’Connor et al., 2007), so we should pay more attention to the people extremely low in spontaneous MTT. Similarly, retrospective bias in mind wandering was often associated with negative mood (Smallwood and O’Connor, 2011). Therefore the relation between the propensities to engage in spontaneous MTT with different temporal focuses and mental health deserves further investigation, and early interventions for abnormal MTT during mind wandering would be valuable. In addition, it is important to recognize the gender and grade differences in the propensity to experience spontaneous MTT (i.e., for girls but not boys, there was a significant increase between 3rd and 4th grade in the past-oriented and future-oriented mind wandering.). Researchers have already provided a comprehensive review of necessary cognitive components for MTT, including working memory, self-awareness, theory of mind, and executive function (Suddendorf and Corballis, 2007). Therefore, the differences described above may reflect different trends in some of these components between gender and age. The key factors in the development of MTT for children will be an important issue for future research.

Study 2 provided evidence for the criterion validity of TFMWQ-C, which showed a significant positive correlation between the trait level and the state level of the temporal focus in spontaneous MTT. At the same time, the significant negative correlation between the frequency of mind wandering during the WMT and task performance suggests that thought sampling can also be appropriate for school-age children (see Mrazek et al., 2013 for related findings). Another intriguing finding was the lack of effect of executive resources on the temporal focus of spontaneous MTT for school-age children. For adults, the prospective bias in mind wandering was curtailed by the requirement to continuously monitor the task (Smallwood et al., 2009b). However, in the current study, the prospective bias of TUTs for children was not influenced by the task demands; that is, the children inclined to experiencing more future-oriented TUTs rather than past-oriented TUTs in both tasks. In order to address this discrepancy further, a better way may be setting a group of tasks with systematically increasing demands (e.g., 1-back, 2-back, and 3-back tasks) and observing the influence of task demands on the temporal focus of TUTs.

To our knowledge, this is the first study to investigate children’s spontaneous MTT during mind wandering. Given that the study of different temporal focuses on children’s mind wandering is in its infancy, there are a number of interesting directions for future research. First, mentally projecting the self forward in time enables a coherent and stable personal identity extending from the past to the future (Tulving, 1985). A growing number of researchers have acknowledged the close relationship between the ability to re-experience the past and simulate the future, and existing developmental and neuroimaging data suggested that thinking about one’s past and future may be similar, but not fully overlapping in cognitive processes (Busby and Suddendorf, 2005; Szpunar et al., 2007). Consistent with this hypothesis, we also found the correlation between the tendencies to prospective and retrospective during mind wandering for children when a big sample was adopted in Study 1. It will be important to further examine the relationship between the mental processes involved in looking into the future and back to the past from a developmental perspective.

The second import direction for future research concerns the relation of mind wandering to the development of theory of mind, based on the assumption that both of them deal with the function of mental simulation. On the one hand, episodic representation is the main way to characterize mind wandering (Song and Wang, 2012). On the other hand, according to the simulation theory of theory of mind (Harris, 1992; Flavell, 2004), children become able to speculate the mental states of other people through a kind of role-taking or simulation process. Our view is that the situational characteristics of mind wandering are likely to provide more opportunities for mental simulation, thus contributing to the development of theory of mind. Our previous research showed that the 4 year old children who reported more experiences of mind wandering during a 3 min resting state had more advanced theory of mind ability (Chen, 2013). Similarly, studies have showed that fantasy assessments are significantly related to the theory of mind performance among preschool children (Taylor and Carlson, 1997). More research on this topic is therefore recommended.

Third, future research could also focus on the functionality of prospective bias during mind wandering in consideration of prospective memory (PM). PM refers to memory for activities to be performed in the future. According to Klinger’s Current Concerns Theory (Klinger, 1999, 2009, 2013), mind wandering is often goal-directed and preparing for the future. If mind wandering possesses the value of anticipation and planning of personally upcoming events, then such properties may be best served by prospective thought during mind wandering (Baird et al., 2011). In the classic study of PM paradigm (Einstein and McDaniel, 1990), subjects often start with a brief distraction task, which aims to avoid the goal of PM tasks being stored in working memory and to generate a certain degree of forgetting, and then they perform the ongoing task embedded with prospective targets. An adapted design could explore the relation between the frequency of prospective thought in distraction/ongoing task and the performance on the PM task. If mind wandering helps one to better maintain PM targets while sacrificing speed or accuracy of distraction/ongoing task, then these lapses of attention could be viewed as instrumental (Cohen, 2013). In our view, situational characterization of mind wandering may promote the intentional encoding of PM as well as strengthen the links between the target and future goals, so as to improve performance on PM. Similarly, some studies have found that age and episodic future thinking abilities were significant predictors of PM performance (Nigro et al., 2013; Neroni et al., 2014). Therefore, future research could shed light on the relationship between the prospective thought during mind wandering and PM from an empirical perspective.

Last but not least, in contrast with the assumption that mind wandering always occurs at a significant cost to task performance, several studies have examined its potential virtues. Studies of fantasy in children have suggested the link between imaginative predisposition and creativity (Taylor, 1997; Singer and Singer, 2008). Interestingly, in the course of our interview, most of the interviewees admitted that the content of their mind wandering sometimes was beneficial to their creative activities (e.g., writing and inventions). The discrepancy in the perspectives to mind wandering may be due to the fact that there is more than one type of cognitive component in mind wandering, and that only some of them play positive roles. Individuals with similar frequencies of mind wandering can have enormous variations in the content and other characteristics of mind wandering. We argue that the study of mind wandering needs to take “deconstructive” strategies, not just “integrative” ones. The good news is that more and more researchers have begun to take this kind of approach (Smallwood, 2013). For instance, from the different temporal focuses (Smallwood et al., 2009b, 2011; Andrews-Hanna et al., 2010; Smallwood and O’Connor, 2011; Stawarczyk et al., 2011; Song and Wang, 2012), from the different emotional tones (Smallwood et al., 2009a; Killingsworth and Gilbert, 2010; Ruby et al., 2013), from the individual differences (McVay and Kane, 2012a,b; McVay et al., 2013), from the relation between mindfulness and mind wandering (Mrazek et al., 2012; Schooler et al., 2014), and so on. One might expect the deconstruction of mind wandering will be the focus of future research.

In conclusion, we extended the study of mind wandering to a child population, developed and validated a questionnaire to measure the individual differences in the temporal focus of spontaneous MTT in daily lives for primary school students, investigated the temporal characteristics of their mind wandering in daily lives and laboratory situations, and suggested some future research directions in this area.

## Conflict of Interest Statement

The authors declare that the research was conducted in the absence of any commercial or financial relationships that could be construed as a potential conflict of interest.
